# Long non-coding RNA-mediated transcriptional interference of a permease gene confers drug tolerance in fission yeast

**DOI:** 10.1038/ncomms6576

**Published:** 2014-11-27

**Authors:** Ryan Ard, Pin Tong, Robin C. Allshire

**Affiliations:** 1 Wellcome Trust Centre for Cell Biology and Institute of Cell Biology, School of Biological Sciences, The University of Edinburgh, Max Born Crescent, Edinburgh EH9 3BF, Scotland, UK

## Abstract

Most long non-coding RNAs (lncRNAs) encoded by eukaryotic genomes remain uncharacterized. Here we focus on a set of intergenic lncRNAs in fission yeast. Deleting one of these lncRNAs exhibited a clear phenotype: drug sensitivity. Detailed analyses of the affected locus revealed that transcription of the *nc-tgp1* lncRNA regulates drug tolerance by repressing the adjacent phosphate-responsive permease gene *transporter for glycerophosphodiester 1* (*tgp1*^*+*^). We demonstrate that the act of transcribing *nc-tgp1* over the *tgp1*^*+*^ promoter increases nucleosome density, prevents transcription factor access and thus represses *tgp1*^*+*^ without the need for RNA interference or heterochromatin components. We therefore conclude that *tgp1*^*+*^ is regulated by transcriptional interference. Accordingly, decreased *nc-tgp1* transcription permits *tgp1*^*+*^ expression upon phosphate starvation. Furthermore, *nc-tgp1* loss induces *tgp1*^*+*^ even in repressive conditions. Notably, drug sensitivity results directly from *tgp1*^*+*^ expression in the absence of the *nc-tgp1* RNA. Thus, transcription of an lncRNA governs drug tolerance in fission yeast.

## Abstract (web summary)

The presence of long non-coding RNAs (lncRNAs) is pervasive across genomes, yet few lncRNAs have clearly established mechanisms of action. Here the authors demonstrate that the fission yeast lncRNA nc-tgp1 regulates expression of the drug tolerance gene *tgp1*_+_ via^+^ transcriptional interference.

## Graphical abstract

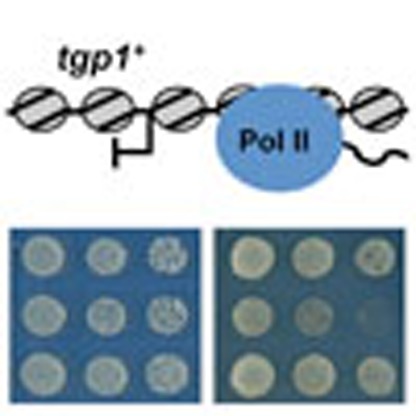

Eukaryotic genomes are pervasively transcribed. Frequently this transcription generates long non-coding RNAs (lncRNAs), which may be transcribed antisense to protein-coding genes, from within introns, or from intergenic regions of the genome. RNA polymerase II (RNAPII) is responsible for generating both messenger RNAs (mRNAs) and lncRNAs^1^. As with mRNAs, many lncRNAs are processed (that is, capped, spliced, polyadenylated), however, in contrast to protein-coding mRNAs, lncRNAs are predominantly nuclear and many are rapidly degraded by the exosome^2^, the major cellular 3′→5′ RNA degradation machinery^3^. Consequently, the majority of lncRNAs exhibit low steady-state levels compared with mRNAs. This instability coupled with their general lack of primary sequence conservation has lead to the suggestion that many lncRNAs might simply result from spurious, inconsequential ‘transcriptional noise’^4^. Nonetheless, accumulating evidence indicates that an increasing number of lncRNAs act to regulate gene expression^2^,^5^.

The mere act of lncRNA transcription, including accompanying chromatin modifications and resulting changes in nucleosome density^6^, can have a profound impact on neighbouring gene expression. In the simplest scenario, lncRNA expression can provide an environment that is either suitable or unsuitable for transcription factor binding. For example, cascading lncRNA transcription upstream of the fission yeast *Schizosaccharomyces pombe fbp1*^*+*^ gene is required to induce *fpb1*^*+*^ expression following glucose starvation^7^. In addition, in a process termed ‘transcriptional interference’, serine-mediated repression of the budding yeast *Saccharomyces cerevisiae SER3* gene is brought about by lncRNA transcription into the gene promoter, which increases nucleosome density and prevents transcription factor access^8^,^9^,^10^. These examples illustrate the positive and negative influence that lncRNA transcription can exert on gene regulation in response to environmental changes.

lncRNAs can also be processed into smaller regulatory RNAs (for example, short interfering RNA)^11^. In *S. pombe*, lncRNAs transcribed from centromeric outer repeats are processed by Dicer (Dcr1) into short interfering RNAs, which target the Clr4 H3K9 methyltransferase via Ago1 (within the RNA-induced transcriptional silencing complex) to establish repressive heterochromatin through the methylation of lysine 9 on histone H3 (refs 12, 13, 14, 15). In addition, lncRNAs may directly associate with and recruit factors that alter chromatin status, in *cis* or in *trans*, silencing genes or behaving as enhancers^16^,^17^. For example, lncRNAs aid the response of *S. cerevisiae* cells to specific changes in nutrient availability by recruiting chromatin-modifying complexes (for example, histone deacetylases) to dynamically regulate several genes^18^,^19^,^20^. Related mechanisms have since been reported in multicellular eukaryotes^21^,^22^. Recent analyses also suggest that patches of transient heterochromatin can form under particular conditions at specific euchromatic loci in *S. pombe*^23^,^24^,^25^. This mechanism involves the RNA-binding protein Mmi1, which recruits the RNA-surveillance machinery to specific determinant of selective removal (DSR) motifs in target transcripts, leading to their exosome-mediated degradation^26^. Mmi1 and its associated factor Red1 are reported to also recruit chromatin-modifying activities via nascent mRNA and lncRNA targets to deposit H3K9 methylation (H3K9me2) at these locations^23^,^25^,^27^,^28^. It is therefore evident that lncRNAs employ a variety of mechanisms to regulate gene expression.

Despite rapid advances in lncRNA identification, only a small number have been characterized in detail. A clear challenge in assigning function has been a lack of lncRNA sequence conservation between even the most closely related species^29^. However, the order of genes flanking the transcription units that encode lncRNAs can be preserved through evolution^30^ (that is, synteny) and provides another criterion by which we can identify potential functionally conserved lncRNAs whose primary sequences might have diverged too much so as not to retain detectable homology.

Only a few of the ~500 annotated intergenic lncRNAs in *S. pombe* are conserved at the sequence level in three divergent *Schizosaccharomyces* species, although many retain synteny with flanking genes in at least one other species^31^. We identified eight discrete intergenic lncRNAs that exhibit synteny in at least three of the four *Schizosaccharomyces* species. Deletion of one of these loci (SPNCRNA.1343 or *ncRNA.1343* for short) exhibited a drug-sensitivity phenotype. We demonstrate that *ncRNA.1343* encodes a bidirectional lncRNA promoter and that its deletion causes loss of expression of the divergent unstable transcript *nc-tgp1*. Our analyses reveal that *nc-tgp1* is targeted for Mmi1-directed exosome degradation and is required to repress a downstream phosphate-responsive gene (SPBC1271.09 designated *transporter for glycerophosphodiester 1 (tgp1*^*+*^)). However, rather than involving transient heterochromatin formation as a result of targeted RNA degradation, the regulation of *tgp1*^*+*^ by the *nc-tgp1* RNA appears to be mediated by transcriptional interference. Most importantly, tolerance of *S. pombe* to a broad spectrum of compounds relies on the regulation of *tgp1*^*+*^ by *nc-tgp1*.

## Results

### Deletion of SPNCRNA.1343 causes drug hypersensitivity

The *S. pombe* genome is predicted to encode ~500 intergenic lncRNAs^32^. Although few of these lncRNAs exhibit detectable sequence conservation, ~100 are conserved in synteny with putative lncRNA orthologues in at least one of the three other known *Schizosaccharomyces* species^31^. For example, the functionally characterized telomerase RNA (*ter1*^*+*^/SPNCRNA.214) is syntenic despite its lack of sequence conservation (see Supplementary Fig. 1a).

To identify other potential functionally conserved lncRNAs, we selected eight lncRNAs, including *ter1*^*+*^ as a control, where surrounding gene order is retained in *S. pombe* and at least two other *Schizosaccharomyces* species. Each lncRNA gene was deleted by replacement with a loxP-flanked *ura4*^+^ marker (Supplementary Fig. 1b). Apart from *ter1Δ*, the selected lncRNAs were not essential for normal cell growth (Supplementary Figs 1c and 2). However, since many characterized lncRNAs regulate gene expression in response to environmental changes and stress^33^, we tested the growth of these lncRNA deletion strains in response to the following stresses: temperature, the microtubule destabilizing drug thiabendazole (TBZ), DNA synthesis inhibitor hydroxyurea (HU), ultraviolet-induced DNA damage, H_2_O_2_-induced oxidative stress and caffeine, an inhibitor of cyclic AMP phosphodiesterase. Cells lacking SPNCRNA.1343 (*ncRNA.1343* for short) displayed a phenotype: hypersensitivity to TBZ, HU and caffeine but not to temperature extremities, ultraviolet-irradiation or oxidative stress (Supplementary Fig. 1c and Supplementary Fig. 2).

### Drug sensitivity of *1343Δ* cells is caused by *tgp1^+^* induction

**Figure 1:**
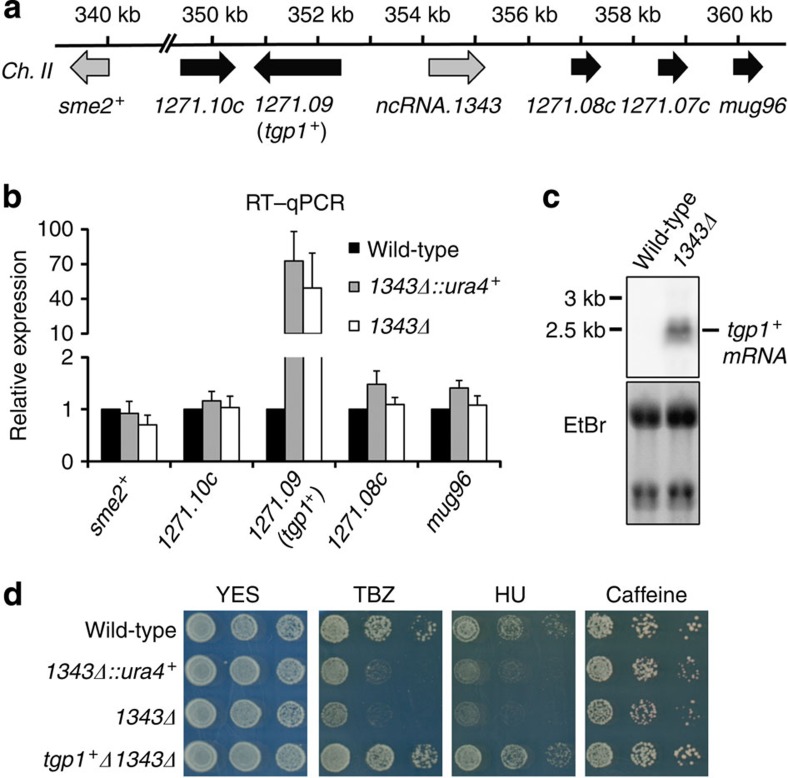
Drug sensitivity following *ncRNA.1343* deletion is due to increased *tgp1*^*+*^ expression. (**a**) Schematic representation of genes flanking *ncRNA.1343*. (**b**) RT–qPCR experiments measured transcript levels for nearby genes in wild-type cells and following replacement of *ncRNA.1343* with *ura4*^*+*^ (*1343Δ::ura4*^*+*^) or deletion (*1343Δ*). Error bars represent s.e.m. resulting from at least three independent replicates. (**c**) Northern analysis of *tgp1*^*+*^ transcript levels in wild-type and *1343Δ* cells grown in the presence of phosphate. (**d**) Serial dilutions of wild-type, *1343Δ::ura4*^*+*^, *1343Δ* and *tgp1Δ1343Δ* double mutant spotted on non-selective YES medium or in the presence of TBZ (20 μg ml^−1^), HU (10 mM) or caffeine (15 mM), respectively.

lncRNAs can act in *cis* to regulate the expression of nearby genes^2^. To determine the cause of drug sensitivity in *1343Δ* cells we examined the expression of genes flanking the locus by real-time quantitative reverse transcriptase-PCR (RT–qPCR) in wild-type cells, cells with *ncRNA.1343* replaced by loxP-flanked *ura4*^*+*^ marker (*1343Δ::ura4*^*+*^) and cells with the *ura4*^*+*^ marker subsequently removed (*1343Δ*; Fig. 1a). SPBC1271.09 transcript levels increased >50-fold in both *1343Δ::ura4*^*+*^ and *1343Δ* cells (Fig. 1b), while the expression of other neighbouring genes was unaltered. SPBC1271.09 encodes a conserved glycerophosphodiester membrane transporter (designated as *tgp1*^+^) orthologous to the *S. cerevisiae* permease *GIT1*. As with *S. cerevisiae GIT1*, the *tgp1*^+^ gene is repressed when cells are grown in the presence of phosphate and induced upon phosphate starvation^34^,^35^. Northern analysis confirmed that *tgp1*^+^ was indeed highly expressed in *1343Δ* cells but not wild-type cells, both grown in the presence of phosphate (repressed condition; Fig. 1c).

To determine whether the drug sensitivity of *1343Δ* cells is a direct result of increased *tgp1*^+^ expression, the *tgp1*^+^ gene was deleted from *1343Δ* cells (*tgp1Δ1343Δ*). This manipulation restored TBZ, HU and caffeine tolerance to levels comparable with wild-type cells (Fig. 1d). We conclude that increased *tgp1*^+^ expression is directly responsible for the drug-sensitivity phenotype of cells lacking *ncRNA.1343*.

### Bidirectional lncRNA promoter upstream of *tgp1^+^*

**Figure 2:**
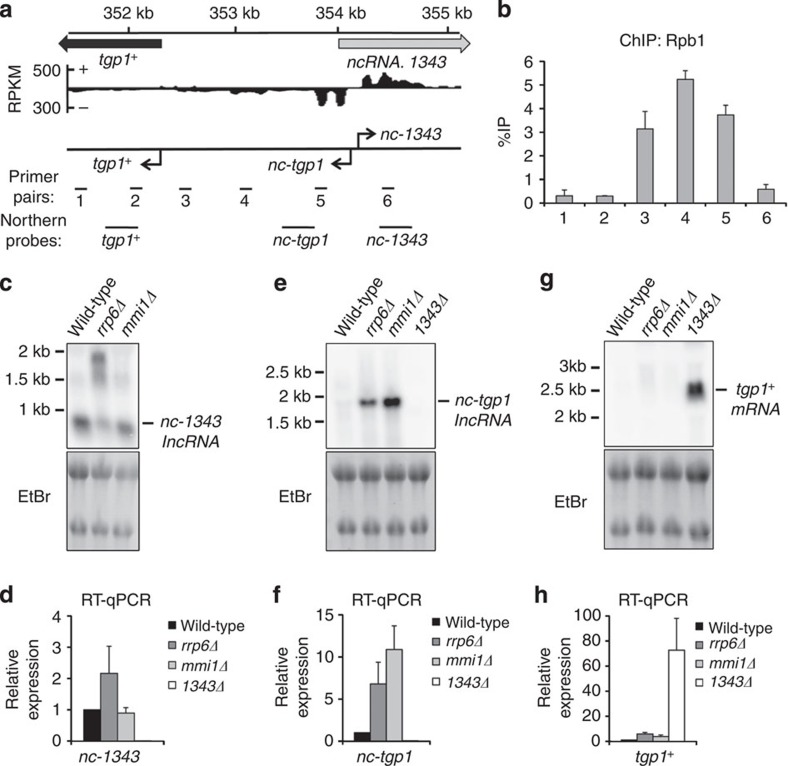
Two distinct lncRNAs are transcribed from a bidirectional promoter upstream of *tgp1*^+^. (**a**) Previously published strand-specific RNA-Seq analysis (Rhind *et al*.,^31^) upstream of SPBC1271.09/*tgp1*^*+*^, represented as reads per kilobase per million (RPKM). Location of qPCR primer pairs and probes for Northern analysis are shown below. (**b**) Rbp1 ChIP–qPCR experiments performed in wild-type cells. (**c**,**e**,**g**) Northern analysis of *nc-1343*, *nc-tgp1* and *tgp1*^*+*^ transcript levels in wild-type, *rrp6Δ*, *mmi1Δ* and *1343Δ*, respectively. (**d**,**f**,**h**) RT–qPCR experiments measured *nc-1343*, *nc-tgp1* and *tgp1*^*+*^ transcript levels in wild-type, *rrp6Δ*, *mmi1Δ* and *1343Δ*, respectively. Error bars represent s.e.m. resulting from at least three independent replicates.

Previous RNA-seq analysis indicates that an lncRNA is transcribed in the sense orientation upstream of *tgp1*^*+*^ (refs 27, 31). We identified two divergent transcriptional start sites arising within *ncRNA.1343*: one lncRNA transcribed towards the *tgp1*^*+*^ gene (*nc-tgp1*) and the other in the opposite orientation (*nc-1343*; Fig. 2a; Supplementary Fig. 3). *lacZ* reporter assays demonstrate that the bidirectional promoter drives greater levels of transcription in the *nc-tgp1* direction (Supplementary Fig. 3). This finding is consistent with Rpb1 Chromatin Immunoprecipitation (ChIP) analysis showing that RNAPII is enriched over the *nc-tgp1* transcription unit, while much lower RNAPII levels are detected on *nc-1343* (Fig. 2b).

We next examined the regulation of the *nc-1343* and *nc-tgp1* transcripts produced from this bidirectional promoter. A ~0.9 kb transcript for *nc-1343* was readily detected in wild-type cells. The size and levels of the *nc-1343* transcript increased in exosome defective (*rrp6Δ*) cells, but not cells lacking Mmi1 or Red1 (Fig. 2c,d; Supplementary Fig. 4). The lncRNA corresponding to *nc-tgp1* was previously detected in *rrp6Δ* and *red1Δ* cells^27^. We identified a consensus DSR motif for Mmi1 binding at position +820 nt within the *nc-tgp1* transcript and RNA IP (RIP) experiments confirmed a direct interaction between Mmi1 and the *nc-tgp1* RNA (Supplementary Fig. 5). Northern analysis identified that an ~1.9 kb *nc-tgp1* RNA accumulates in *rrp6Δ*, *mmi1Δ* and *red1Δ*, but not in wild-type cells (Fig. 2e,f; Supplementary Fig. 4). Interestingly, a recent study found that the repressive lncRNA transcribed upstream of the phosphate-responsive *pho1*^*+*^ gene in *S. pombe* also contains a DSR motif and is targeted by Mmi1 for exosome-mediated degradation^28^, indicating that a similar regulatory mechanism might control expression of *tgp1*^*+*^ and *pho1*^*+*^. In sum, both *nc-1343* and *nc-tgp1* transcripts are processed by the exosome, but only *nc-tgp1* is regulated by Mmi1-mediated recruitment of the nuclear exosome.

A moderate increase in *tgp1*^*+*^ transcript levels has previously been reported in cells lacking Mmi1 (ref. 23). In agreement with this, we detected a similar increase (approximated four-fold) in *tgp1*^*+*^ transcript levels in *mmi1Δ* or exosome (*rrp6Δ* or *dis3-54*) mutant cells by RT–qPCR, however, this increase is significantly less than the >50-fold upregulation of *tgp1*^*+*^ observed in *1343Δ* cells (Fig. 2g,h; Supplementary Fig. 4). Moreover, we failed to detect the *tgp1*^*+*^ transcript in *rrp6Δ* or *mmi1Δ* cells by Northern analysis, indicating that *tgp1*^*+*^ is not induced in the absence of these factors. Thus, Mmi1-mediated exosome degradation is not the predominant mechanism involved in *tgp1*^*+*^ regulation.

### *tgp1^+^* is repressed by the *nc-tgp1* lncRNA

**Figure 3:**
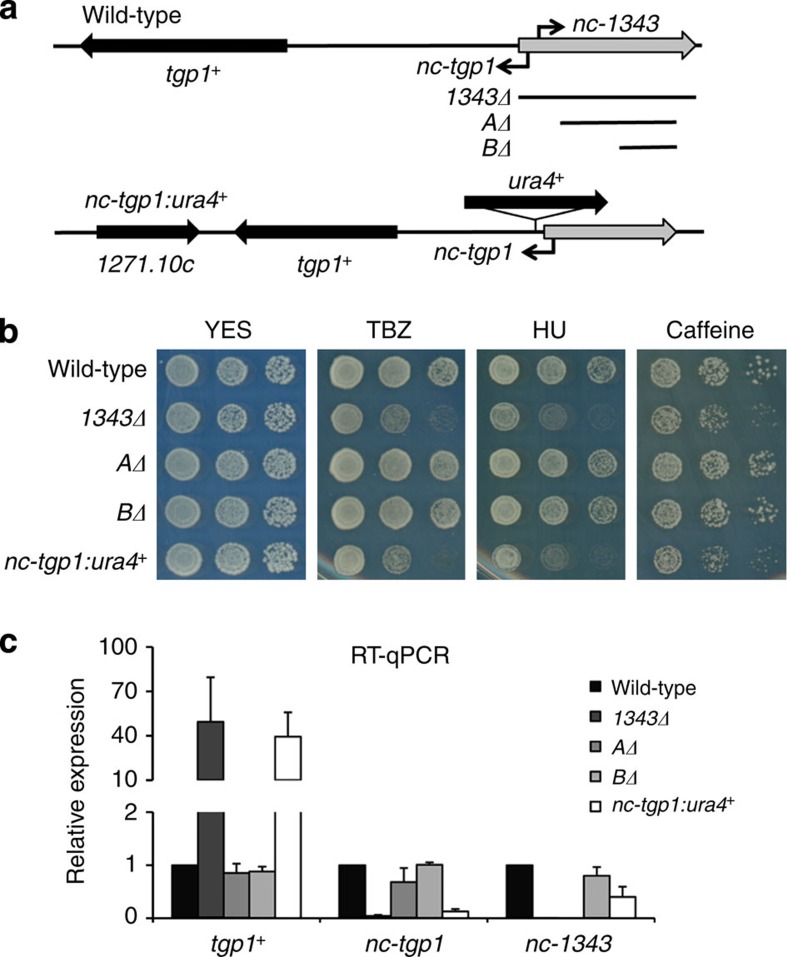
*nc-tgp1*, not *nc-1343*, represses *tgp1*^*+*^ to confer drug tolerance. (**a**) Schematic diagram indicating strategic manipulations of lncRNAs upstream of *tgp1*^*+*^ including *1343Δ*, shorter deletions of *ncRNA.1343* (*AΔ* and *BΔ*) and *ura4+* integration within the *nc-tgp1* lncRNA locus (*nc-tgp1:ura4*^*+*^) in wild-type background. (**b**) Serial dilutions of wild-type, *1343Δ, AΔ*, *BΔ* and *nc-tgp1:ura4*^*+*^ were spotted on non-selective YES medium or in the presence of TBZ (20 μg ml^−1^), HU (10 mM) or caffeine (15 mM), respectively. (**c**) RT–qPCR experiments measured *tgp1*^*+*^, *nc-tgp1* and *nc-1343* transcript levels in wild-type, *1343Δ, AΔ*, *BΔ* and *nc-tgp1:ura4*^*+*^ cells. Error bars represent s.e.m. resulting from three independent replicates.

The presence of the unstable *nc-tgp1* RNA upstream of *tgp1*^*+*^ suggests that either *nc-tgp1*, *nc-1343* or both regulate *tgp1*^*+*^ expression. To test the involvement of these lncRNAs in *tgp1*^*+*^ regulation, a series of strategic genetic manipulations were performed (Fig. 3a). Truncations of *nc-1343* (that is, *AΔ* and *BΔ*) that retain its 5′ end did not result in the drug-sensitivity phenotype presented by *1343Δ* cells (Fig. 3b) and, similarly, did not induce *tgp1*^*+*^ expression (Fig. 3c). This indicates that full-length *nc-1343* is not required for *tgp1*^*+*^ repression. We next tested if *nc-tgp1* is involved in repressing *tgp1*^*+*^. Our analyses show that transcription of *nc-tgp1* starts within the encoded *ncRNA.1343* transcription unit (Supplementary Fig. 3). Thus, deletion of the entire locus (*1343Δ*) removes the *nc-tgp1* promoter, and the 5′ end of its transcript, resulting in the observed loss of *nc-tgp1* expression (Figs 2f and 3c). The *AΔ* and *BΔ* truncations of *nc-1343*, which retain the *nc-tgp1* promoter, do not affect *nc-tgp1* transcription or relieve repression of *tgp1*^*+*^. In contrast, interruption of the *nc-tgp1* transcription unit by insertion of the *ura4*^*+*^ marker gene (*nc-tgp1:ura4*^*+*^) prevented *nc-tgp1* transcription, induced *tgp1*^*+*^ expression to levels observed in *1343Δ* levels and increased sensitivity of these cells to TBZ, HU and caffeine (Fig. 3b,c). These analyses demonstrate that it is *nc-tgp1*, not *nc-1343*, that is critical for repressing *tgp1*^*+*^ in the presence of phosphate.

### Phosphate starvation induces *tgp1^+^* by repressing *nc-tgp1*

**Figure 4:**
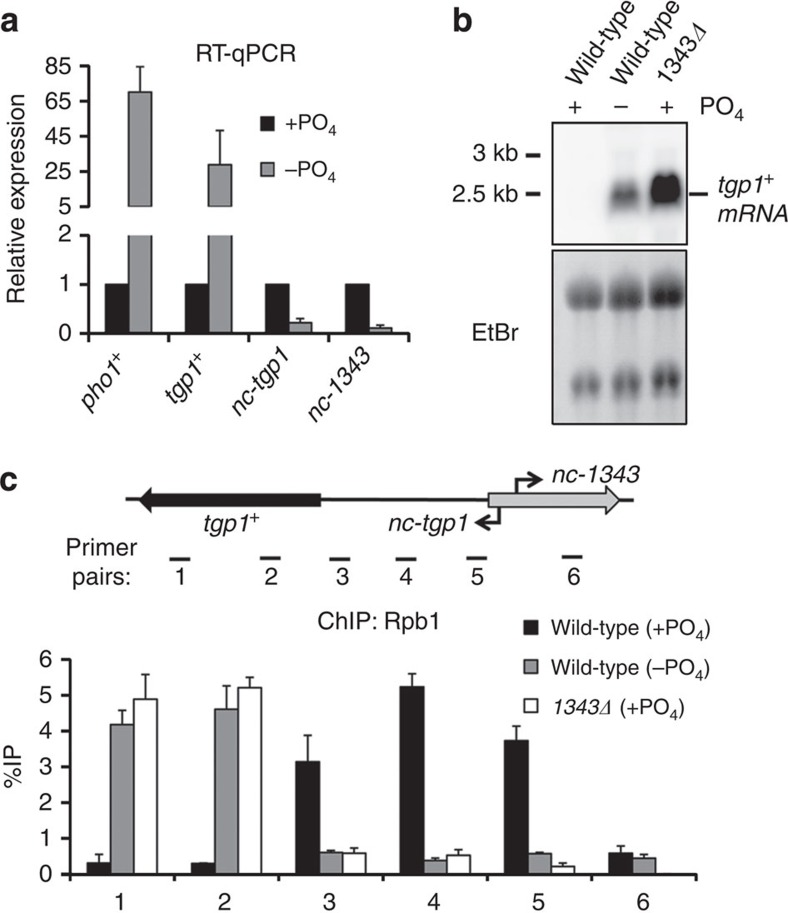
Phosphate starvation induces *tgp1*^*+*^ and reduces lncRNA transcription. (**a**) RT–qPCR experiments measured *tgp1*^*+*^, *nc-tgp1* and *nc-1343* transcript levels in wild-type cells grown in phosphate-rich medium (+PO_4_) or in the absence of phosphate (−PO_4_). *pho1*^*+*^ is a positive control for phosphate starvation. (**b**) Northern analysis of *tgp1*^*+*^ in wild-type cells grown in the presence or absence of phosphate and *1343Δ* grown in the presence of phosphate. (**c**) Rbp1 ChIP–qPCR experiments performed in wild-type cells grown in the presence or absence of phosphate and *1343Δ* grown in the presence of phosphate. Error bars represent s.e.m. resulting from three independent replicates.

Upon phosphate starvation of fission yeast, several genes involved in the phosphate response are induced (including *tgp1*^*+*^ and *pho1*^*+*^) (ref. 35). To determine how the transcription of *nc-tgp1* is altered in response to phosphate and how it might influence *tgp1*^*+*^ expression we assessed expression in phosphate-rich (+PO_4_) and phosphate-deprived (−PO_4_) conditions. As expected, the levels of *tgp1*^*+*^ and the *pho1*^*+*^ control increased upon phosphate starvation (Fig. 4a,b). In contrast, the levels of both *nc-tgp1* and *nc-1343* RNAs decreased significantly in the absence of phosphate (Fig. 4a; Supplementary Fig. 6). The observed reduction in *nc-tgp1* levels is consistent with a situation whereby loss or reduction of *nc-tgp1* transcription permits *tgp1*^*+*^ induction. In agreement with this, significantly less RNAPII associates with the *nc-tgp1* transcription unit in both phosphate-starved wild-type cells and phosphate-replete *1343Δ* cells, which do not transcribe *nc-tgp1* (Fig. 4c). Therefore, preventing *nc-tgp1* transcription, even in phosphate-rich medium, recapitulates the changes in RNAPII occupancy that normally accompany *tgp1*^*+*^ induction upon phosphate deprivation.

### RNAi-directed heterochromatin does not repress *tgp1^+^*

**Figure 5:**
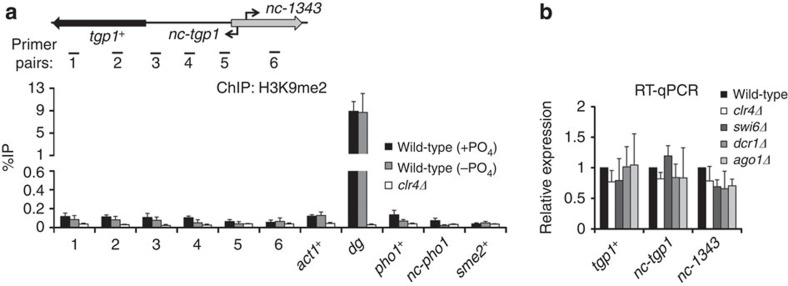
*tgp1*^*+*^ is not regulated by RNAi/heterochromatin. (**a**) H3K9me2 ChIP–qPCR experiments performed in the presence or absence of phosphate. *clr4Δ* was used as a negative control. The euchromatic actin gene (*act1*^*+*^) and centromeric *dg* repeats (*dg*) are positive and negative controls for heterochromatin. *pho1*^*+*^ is a phosphate-regulated gene repressed by *nc-pho1*, a lncRNA target of Mmi1. *sme2*^*+*^ is another lncRNA target of Mmi1. H3K9me2 to bulk H3 ratio has not been presented due to background methyl H3K9 levels detected at these loci. (**b**) RT–qPCR experiments measured *tgp1*^*+*^, *nc-tgp1* and *nc-1343* transcript levels in wild-type cells and cells lacking factors involved in heterochromatin formation and stability, respectively. Error bars represent s.e.m. resulting from at least three independent replicates.

Cells with defective exosome function (*rrp6Δ*) accumulate non-coding RNAs, some of which have been reported to attract Mmi1-dependent RNA elimination factors, along with RNA interference (RNAi) components and the Clr4 H3K9 methyltransferase, leading to the formation of transiently regulated HOODs (heterochromatin domains)^25^. The regions containing the *tgp1*^*+*^ and *pho1*^+^ genes are included in HOOD-17 and HOOD-24, respectively, and both form a region of Mmi1-directed transient heterochromatin in *rrp6Δ* cells^24^,^27^. The *nc-tgp1* transcript is clearly regulated by Mmi1-directed exosome degradation (Fig. 2e,f), however, we do not detect methylated H3K9 (H3K9me2) over the *tgp1*^*+*^, *nc-tgp1* or *nc-1343* genes within HOOD-17 in wild-type cells (Fig. 5a). Likewise, only very low levels of H3K9me2, slightly above background in cells lacking the H3K9 methyltransferase (*clr4Δ*), could be detected on the *pho1*^*+*^ gene and the upstream Mmi1-targeted lncRNA (*nc-pho1*) within HOOD-24. Moreover, this low level of H3K9me2 did not drop appreciably upon induction of *tgp1*^*+*^ and *pho1*^+^ (−PO_4_; Fig. 5a). Equivalent background levels of H3K9me2 were detectable on another Mmi1-targeted lncRNA gene (*sme2*^+^) and the highly expressed actin gene (*act1*^+^). In contrast, H3K9me2 was ~100-fold enriched over the centromeric outer repeats (*dg*) in wild-type cells, but reduced to background in *clr4Δ* cells, indicating that H3K9-methylated chromatin had been efficiently immunoprecipitated. In addition, the transcript levels of *tgp1*^*+*^, *nc-tgp1*, *nc-1343*, *pho1*^+^ and *nc-pho1* were unaffected by loss of RNAi (for example, *ago1Δ* or *dcr1Δ*) or heterochromatin components (for example, *clr4Δ* or *swi6Δ*) (Fig. 5b; Supplementary Fig. 7a). Nor were the kinetics of *tgp1*^*+*^ or *pho1*^+^ induction following phosphate starvation altered in cells lacking heterochromatin (Supplementary Fig. 7b,c). In contrast, *nc-tgp1*, *nc-pho1* and *sme2*^*+*^ RNA levels were clearly elevated in cells lacking Mmi1-mediated exosome degradation (*mmi1Δ* and *rrp6Δ*). Thus, although H3K9me2 accumulates at particular regions in *rrp6Δ* cells (for example, HOOD-17: *tgp1*^*+*^ and HOOD-24: *pho1*^*+*^), we conclude that RNAi and heterochromatin play no appreciable role in regulating these genes under normal physiologically repressive conditions or during their induction.

### *nc-tgp1* prevents Pho7 transcription factor binding

**Figure 6:**
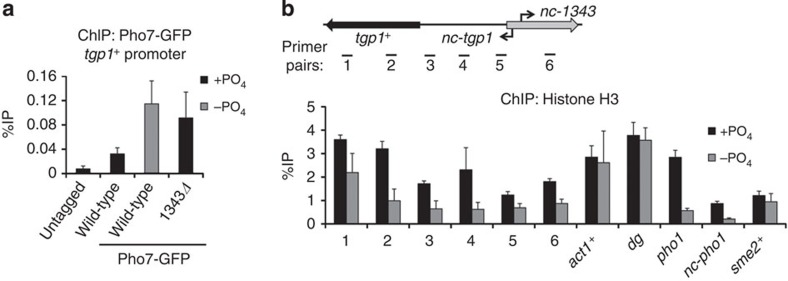
*nc-tgp1* transcription prevents stable Pho7 binding and increases nucleosome density upstream of *tgp1*^+^. (**a**) GFP ChIP–qPCR experiments were performed in the presence or absence of phosphate in cells with C-terminally GFP-tagged Pho7. An untagged strain was used as a negative control. Primer pair #3 was used to detect Pho7 binding at the *tgp1*^*+*^ promoter. (**b**) Nucleosome density was measured by histone H3 ChIP–qPCR experiments in wild-type cells grown in the presence or absence of phosphate. Error bars represent s.e.m. resulting from three independent replicates.

The above analyses indicate that *nc-tgp1* is transcribed into the *tgp1*^*+*^ promoter and suggest that production of this upstream lncRNA represses *tgp1*^*+*^ expression. We therefore investigated if transcription of *nc-tgp1* interferes with the induction mechanism of *tgp1*^*+*^ in response to phosphate starvation. The Pho7 transcription factor has previously been shown to engage phosphate-response gene promoters in phosphate-starved cells^35^,^36^. Our ChIP analyses confirmed that Pho7–green fluorescent protein (Pho7–GFP) accumulates on the *pho1*^*+*^ promoter in phosphate-depleted cells (Supplementary Fig. 8). In addition, Pho7–GFP levels accumulate over the region upstream of *tgp1*^*+*^ when activated (Fig. 6a). However, in cells unable to transcribe *nc-tgp1* (*1343Δ*), higher levels of Pho7–GFP associate with the region upstream of *tgp1*^*+*^ even in repressive conditions (that is, +PO_4_). We conclude that loss of *nc-tgp1* expression due to phosphate starvation or by preventing production of this lncRNA (for example, *1343Δ*) allows Pho7 binding and subsequent *tgp1*^*+*^ induction.

Active RNAPII promoters display reduced nucleosome density^37^. lncRNA transcription over promoters can increase nucleosome density and prevent gene induction^8^,^10^,^20^. We found that histone H3 levels were greater over the *tgp1*^*+*^ gene and upstream region when it is repressed (+PO_4_) compared with when it is expressed (−PO_4_; Fig. 6b). In contrast, H3 levels over control loci (*act1*^*+*^, *sme2*^*+*^ and *dg* repeats) were unaffected by phosphate availability. Thus, upstream transcription appears to alter nucleosome density over the *tgp1*^*+*^ promoter and thereby occlude Pho7 binding. Likewise, a considerable drop in H3 levels was observed on the *pho1*^+^ gene and *nc-pho1* lncRNA region upstream in phosphate-poor conditions, implying a similar mechanism may also operate to regulate the expression of *pho1*^*+*^. We conclude that transcription of the upstream lncRNA inhibits expression of *tgp1*^*+*^ by a transcriptional interference mechanism that alters the chromatin landscape, preventing access to the key phosphate-responsive transcription factor Pho7.

**Figure 7:**
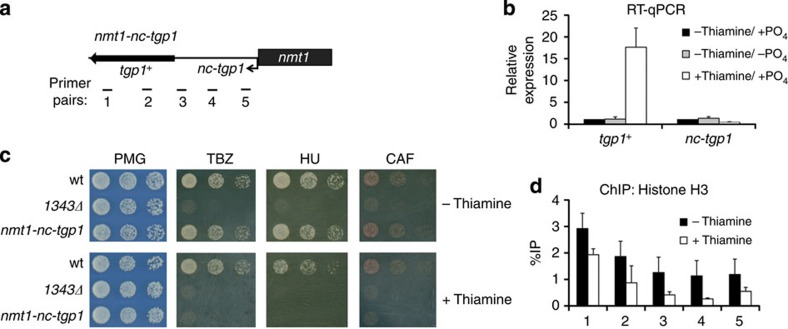
*nmt1* controlled *nc-tgp1* alters drug tolerance in response to thiamine. (**a**) Schematic diagram of *nc-tgp1* under the control of the strong, thiamine-repressible *nmt1* promoter. (**b**) RT–qPCR experiments measured *tgp1*^*+*^ and *nc-tgp1* levels in response to thiamine and phosphate availability using *nmt1-nc-tgp1* cells. (**c**) Serial dilutions of wild-type, *1343Δ* and *nmt1-nc-tgp1* cells were spotted on non-selective PMG medium or in the presence of TBZ, HU or caffeine, respectively, with or without thiamine as indicated. (**d**) H3 ChIP–qPCR experiments in *nmt1-nc-tgp1* cells grown in the presence or absence of thiamine. Error bars represent s.e.m. resulting from three independent replicates.

**Figure 8:**
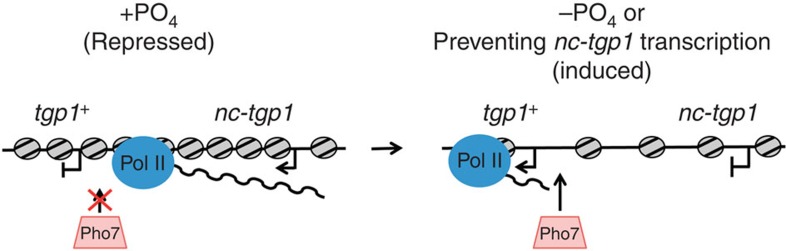
Model for transcriptional interference at *tgp1*^*+*^. The presence of phosphate induces transcription of an unstable lncRNA (*nc-tgp1*). lncRNA transcription increases nucleosome density, occludes Pho7 transcription factor binding and thus represses *tgp1*^*+*^ expression. *nc-tgp1* transcription is reduced following phosphate starvation, decreasing nucleosome density, allowing Pho7 to stably engage the *tgp1*^*+*^ promoter and induce *tgp1*^*+*^ expression.

To directly test if transcriptional interference of *tgp1*^*+*^ by *nc-tgp1* is responsible for *tgp1*^*+*^ repression, we replaced the *nc-tgp1* promoter with the strong, thiamine-regulated *nmt1* promoter (*nmt1-nc-tgp1*) (Fig. 7a). Transcription of *nc-tgp1* from the *nmt1* promoter is rendered unresponsive to phosphate. Instead, *nc-tgp1* is repressed or derepressed in the presence or absence of thiamine, respectively. When *nc-tgp1* was transcribed from the *nmt1* promoter, *tgp1*^*+*^ remained repressed regardless of phosphate availability (Fig. 7b). In contrast, repression of *nmt1*-driven *nc-tgp1* by thiamine resulted in the induction of *tgp1*^*+*^ expression in phosphate-rich media and consequently caused drug sensitivity (Fig. 7b,c). In addition, H3 levels over the region upstream of *tgp1*^*+*^ were high when *nc-tgp1* was transcribed and reduced when *nc-tgp1* was repressed by thiamine (Fig. 7d). Lastly, exogenous expression of full-length *nc-tgp1* from a plasmid failed to repress *tgp1*^*+*^, ruling out the possibility that *nc-tgp1* operates in *trans* (Supplementary Fig. 9). Collectively, these findings confirm that it is the transcription of *nc-tgp1* over the *tgp1*^*+*^ promoter that alters nucleosome density to regulate *tgp1*^*+*^ induction (see Fig. 8) and, as a consequence, drug tolerance of fission yeast cells.

## Discussion

An increasing number of lncRNAs have been shown to tightly regulate eukaryotic gene expression following intra-/extra-cellular environment changes that require rapid, integrated responses at the level of transcription^2^. In *S. pombe*, for example, the balance of antisense lncRNAs and sense transcription controls various stress-response pathways^33^,^38^. However, little is known about the majority of *S. pombe* intergenic lncRNAs. Here we selected and deleted eight stable, discrete lncRNAs in *S. pombe* that show conserved synteny in at least two of the three other known *Schizosaccharomyces* species. Excluding the *ter1*^*+*^ control, only deletion of *ncRNA.1343* exhibited a definitive phenotype: sensitivity to various compounds due to induction of a nearby phosphate-responsive permease gene (*tgp1*^*+*^). Closer inspection revealed that the *ncRNA.1343* promoter is bidirectional. Furthermore, transcription from this bidirectional promoter favours the production of a previously unannotated and unstable lncRNA (*nc-tgp1*) towards the *tgp1*^*+*^ gene under repressive conditions.

Recent studies in fission yeast have implicated lncRNAs in directing repression of specific genes by a mechanism involving transient RNAi-dependent heterochromatin formation^27^. For example, the Mmi1-targeted lncRNA upstream of *pho1*^*+*^ has recently been reported to recruit RNAi-directed heterochromatin to repress *pho1*^*+*^ in response to phosphate availability^28^. However, these findings differ from genome-wide H3K9me2 mapping which show that *tgp1*^*+*^ and *pho1*^*+*^, both of which are regulated by upstream lncRNAs that are targeted for exosome-mediated degradation by Mmi1 (Fig. 2; ref 28), only accumulate RNAi-directed H3K9me2 in mutants with defective RNA processing/degradation (for example, *rrp6Δ*) and not in wild-type cells grown under repressive phosphate-rich conditions^24^. The significance of *rrp6Δ*-dependent heterochromatin at the *tgp1*^*+*^ and *pho1*^*+*^ genes is therefore unclear. Cells lacking Rrp6 accumulate aberrant RNAs and exhibit disrupted heterochromatin globally, including significantly decreased H3K9me2 over centromeric repeats^39^. Therefore caution must be exercised when interpreting the analyses of mutants with such severe defects in RNA processing/degradation. Importantly, we do not detect significant levels of H3K9me2 enrichment on the *tgp1*^*+*^ and *pho1*^*+*^ promoters/genes in wild-type cells under repressive (phosphate-rich) conditions. We cannot exclude the possibility that distinct assay conditions in a previous report allowed detection of low H3K9me2 levels on the *pho1*^*+*^ promoter when repressed^28^, however, the consequence of such H3K9me2 remains uncertain given that our analyses show that the expression of *pho1*^*+*^ or *tgp1*^*+*^ is unaffected by loss of RNAi/heterochromatin. We note that our findings are in agreement with previous expression profiling analyses, showing unaltered *tgp1*^*+*^ and *pho1*^*+*^ levels in *S. pombe* cells lacking RNAi/heterochromatin^40^. In contrast, transcripts arising from *bone fide* heterochromatin in centromeric outer repeats are clearly elevated when RNAi/heterochromatin is defective. Thus, our analyses indicate that the repression of both *tgp1*^*+*^ and *pho1*^*+*^ is unlikely to involve regulated heterochromatin in wild-type cells. Instead, we favour a model whereby *tgp1*^*+*^ and *pho1*^*+*^ are repressed by a transcriptional interference mechanism.

Transcriptional interference is well-established in many systems. In the bacterium *Escherichia coli*, the gene encoding the *clr* transcriptional activator is repressed in response to nitrogen starvation by the act of lncRNA transcription from an alternate upstream promoter^41^. In the single celled eukaryote *S. cerevisiae*, which lacks RNAi and heterochromatin, transcription of the *SRG1* lncRNA into the *SER3* promoter, or heterologous promoters, was found to alter nucleosome density and interfere with transcription factor binding^8^,^9^,^10^. Similarly, in *S. cerevisiae*, non-coding transcription over the *IME1* (ref. 20), *GAL7* (ref. 42) and *FLO11* (ref. 43) promoters prevent gene induction. Analogous mechanisms have also been reported in multicellular eukaryotes. For example, the *Drosophila Ubx* gene^44^, the human dihydrofolate reductase gene^45^ and the imprinted *Igf2r* gene in mammals^46^ are repressed independent of RNAi or transient heterochromatin formation by non-coding transcription into their respective promoters. These examples illustrate that transcriptional interference is a simple, conserved mechanism for modulating specific genes without requiring additional *trans*-acting regulatory factors. Our results are consistent with both *nc-tgp1* and *nc-pho1* mediating repression of downstream genes (*tgp1*^*+*^ and *pho1*^*+*^, respectively) by transcriptional interference, not by the formation of transient heterochromatin. We base this conclusion on our findings that: (i) *tgp1*^*+*^ and *pho1*^*+*^ expression is unaffected by loss of RNAi/heterochromatin; (ii) H3K9me2 is not associated with *tgp1*^*+*^ or *pho1*^*+*^ loci in wild-type cells; (iii) *nc-tgp1* transcription declines when *tgp1*^*+*^ is induced (−PO_4_); (iv) loss of the *nc-tgp1* transcript allows induction of *tgp1*^*+*^ under normally repressive (+PO_4_) conditions (similarly, loss of lncRNA transcription upstream induces *pho1*^*+*^ in repressive medium^27^,^28^); (v) transcription of *nc-tgp1* by a thiamine-repressible promoter brings *tgp1*^*+*^ under the control of thiamine, rather than phosphate; (vi) RNAPII and nucleosome density is increased over the *tgp1*^*+*^ promoter region when the repressive *nc-tgp1* RNA is transcribed and (vii) the Pho7 activator binds the *tgp1*^*+*^ promoter region when *nc-tgp1* transcription is lost.

Genome-wide RNA sequencing has allowed the detection of a large number of lncRNAs in a variety of species. However, it remains unclear how many of these lncRNA are functional transcripts that act to influence gene expression and/or chromatin landscapes. Examples such as Xist RNA in mammals and roX RNAs in *Drosophila* represent functional transcripts that are critical for mediating dosage compensation by altering chromatin status and expression levels from sex chromosomes^47^. However, enthusiasm for lncRNA function has been somewhat dampened by reports showing that the ablation in animal models of some of the best-characterized lncRNAs (for example, HOTAIR, MALAT1, Kcnq1ot1, NEAT1) exhibited less dramatic or undetectable phenotypes^48^,^49^,^50^,^51^,^52^,^53^. Of the discrete stable lncRNAs that we deleted in fission yeast, only one (*ncRNA.1343*) had an obvious phenotype in the growth conditions tested. Detailed analysis was required to reveal that deletion of *ncRNA.1343* actually affected expression of a divergent unstable lncRNA (*nc-tgp1*) transcribed in the opposite orientation as the annotated locus. Only after further manipulation and analyses could we conclude that the expression of *nc-tgp1* interferes with the expression of *tgp1*^*+*^ downstream. The fact that the unstable *nc-tgp1* transcript is the functional partner of the apparently non-functional stable *nc-1343* RNA transcribed from the same bidirectional promoter demonstrates the importance of comprehensive analyses of ncRNAs and the consequences of their deletion. Based on our analyses. we surmise that the low level expression of *nc-1343* represents transcriptional noise, resulting as a byproduct of ample *nc-tgp1* transcription. The syntenic conservation of *ncRNA.1343* within the *Schizosaccharomyces* genus^31^ hints at the possibility of a conserved regulatory mechanism that involves lncRNA transcription into the promoter region of *tgp1*^*+*^ in related species. Thus, although genome-wide approaches can rapidly catalogue the presence and response of various lncRNAs to different conditions, much more detailed locus-specific analyses is required to pinpoint the function of each individual lncRNA with respect to *cis* regulation of nearby genes or *trans* regulation of genes at distal loci.

## Methods

### Yeast strains, plasmids and standard techniques

*S. pombe* strains used in this study are listed in Supplementary Table 1. Standard methods were used for fission yeast growth, genetics and manipulations^54^. All strains were grown in Yeast extract plus supplement medium (YES), unless otherwise indicated. For phosphate starvation experiments, cells were grown to mid-log phase in YES medium, washed twice in dH_2_O, and then grown for indicated times in Pombe minimal glutamate (PMG) synthetic medium without Na_2_HPO_4_ (−PO_4_). Genetic deletions and protein tagging were carried out by lithium acetate transformation. All genetic modifications were confirmed by colony PCR. Plasmids were transformed by electroporation. Selections were performed on PMG/agar plates with according auxotrophy or on YES/agar plates with appropriate antibiotic(s) and grown at 32 °C. Serial (1:4) dilutions of equal number of cells were spotted onto YES/agar and grown at 32 °C, unless indicated otherwise. For drug-sensitivity experiments, cells were spotted onto YES/agar or PMG/agar with DMSO or TBZ (20 μg ml^−1^), HU (10 mM), caffeine (15 mM) and H_2_O_2_ (1 mM). For ultraviolet-sensitivity experiments, spotted cells were ultraviolet-irradiated at 80 J m^−2^ with a Stratalinker UV Crosslinker and grown in the dark at 25 °C. The plasmids containing *lacZ* under the control of the *nc-tgp1* and *nc-1343* bidirectional promoter were cloned as follows. The non-coding promoter was amplified from *S. pombe* genomic DNA in both orientations (using lacZ_1_F/lacZ_1_R and lacZ_2_F/lacZ_2_R primer pairs; see Supplementary Table 2) and ligated into pREP vector containing *lacZ* using PstI/SalI restriction sites. To test if *nc-tgp1* can repress *tgp1*^*+*^ in *trans*, the *nc-tgp1* transcription unit was amplified from *S. pombe* genomic DNA (using nc-tgp1_SalI_F and nc-tgp1_XmaI_R primer pairs, see Supplementary Table 2) and ligated into pREP3x using SalI/XmaI restriction sites.

### Liquid assay for β-galactosidase activity

Assays for *β-galactosidase* activity were performed as described^55^. Briefly, yeast containing vectors expressing *lacZ* under the control of various promoters were grown to log phase (OD_595_ of ~0.5) in selective media. Cells were permeabolized by SDS/chloroform. Cell extracts were equilibrated at 30 °C for 5 min before the addition of ortho-Nitrophenyl-β-galactoside (ONPG). The reaction was stopped with Na_2_CO_3_ once the solution turned yellow and elapsed time was recorded. Cell debris was spun and the OD_420_ was measured. Units were calculated as follows: Units/OD=1000 × (OD_420_/Volume × Time × OD_595_).

### Chromatin and RIP

Cells were grown to mid-log phase at 32 °C in YES. For phosphate starvation experiments, cells in mid-log phase were washed twice in dH_2_O before being grown in PMG (−PO_4_) for 4 h. ChIP was performed essentially as described^12^. Briefly, cells were fixed with 1% paraformaldehyde for 15 min at room temperature. Cells were lysed by bead beating (Biospec Prodcutes) and sonicated using a Bioruptor (Diagenode) sonicator at 5 °C on high for a total of 20 min (30 s ON/OFF cycles). Five microlitres of Rpb1 antibody (#2629; Cell Signaling), 2 μl GFP antibody (G10362; Life Technologies), 2 μl H3 antibody (ab1791; Abcam) and 1 μl of H3K9me2 antibody (m5.1.1; ref. 56) were used for IPs. RIP experiments were performed essentially as described^13^. Hisx6-TEV-Protein A-tagged Mmi1 was captured from cell lysate with IgG Dynabeads (Life Technologies). Mmi1-bound RNA was isolated by phenol-chloroform extraction, DNase treated and reverse transcribed. Quantitative analysis was performed by qPCR.

### RNA analysis

RNA was isolated from *S. pombe* using RNeasy Mini- or Midi-Kits as per manufacturer’s instructions (Qiagen). For RT–qPCR experiments, first strand complementary DNA synthesis was performed on Turbo DNase (Life Technologies) treated RNA using random hexamers and Superscript III (Invitrogen) as per manufacturer’s instructions. Negative controls lacking RT were performed alongside all RT–qPCR experiments. Northern analysis of long non-coding transcripts was performed using UTP-[a^32^P]-labelled RNA probes as described^57^. Transcription start sites were mapped using the SMARTer RACE complementary DNA Amplification Kit as per manufacturer’s instructions (Clontech).

### Quantitative real-time PCR

Primers used in this study are listed in Supplementary Table 2. qPCR was performed using SYBR Green on a Roche Lightcycler. Data was analyzed with LightCycler 480 Software 1.5.0.39. RT–qPCR levels were calculated by normalizing product of interest to an internal reference gene (*act1*^+^). Expression levels were expressed relative to levels detected in wild-type cells. ChIP enrichments were calculated as the ratio of product of interest from IP sample normalized to the corresponding input sample and expressed as ‘%IP’. Error bars represent s.e.m., resulting from at least three independent replicates.

## Additional information

**How to cite this article:** Ard, R. *et al*. Long non-coding RNA-mediated transcriptional interference of a permease gene confers drug tolerance in fission yeast. *Nat. Commun.* 5:5576 doi: 10.1038/ncomms6576 (2014).

## Supplementary Material

Supplementary Information Supplementary Figures 1-9 and Supplementary Tables 1-2
