# Supplementary material for: Long non-coding RNA-mediated transcriptional interference of a permease gene confers drug tolerance in fission yeast
Source: Nat Commun. 2014 Nov 27;5:5576. doi: 10.1038/ncomms6576 (PMC4255232; doi:10.1038/ncomms6576)
Supplement: Supplementary Information — Supplementary Figures 1-9 and Supplementary Tables 1-2 [file ncomms6576-s1.pdf]

Ard_Supplementary-Figure-1

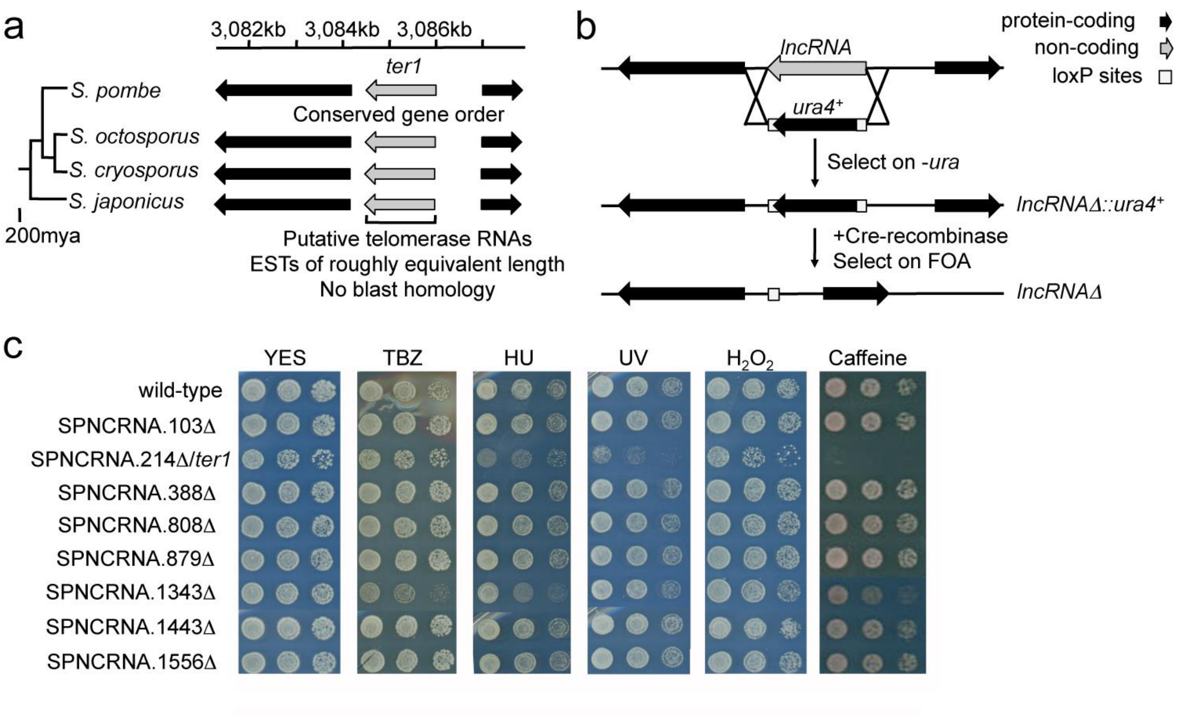

Supplementary Figure 1. Deletion of the lncRNA locus SPNCRNA.1343 results in sensitivity to multiple drugs. (a) Schematic representation of *S. pombe* lncRNA (in this case: SPNCRNA.214, telomerase RNA or *ter1*) with conserved synteny in related *Schizosaccharomycs* species. (b) Schematic diagram of the strategy employed to delete lncRNAs in *S. pombe*. (c) Serial dilutions of lncRNA deletions were spotted on non-selective YES medium or in the presence of various stresses, including exposure to the microtubule destabilizing drug thiabendazole (TBZ; 20 μg/mL), DNA synthesis inhibitor hydroxyurea (HU; 10 mM), UV-irradiation (80 J/m^2), oxidative stress (H_2O_2; 1 mM), or caffeine (15 mM).

Ard_Supplementary-Figure-2

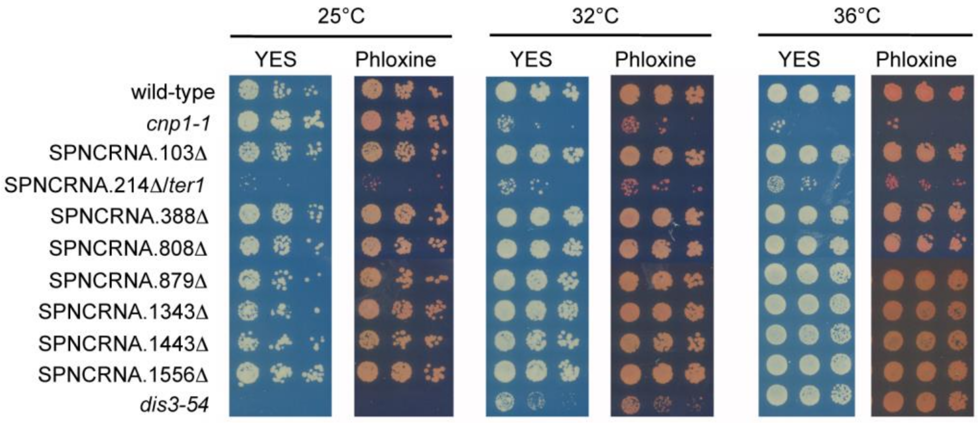

Supplementary Figure 2. lncRNA deletions, excluding *ter1*⁺ control, are viable at 25, 32, and 36°C. Serial dilutions of lncRNA candidate deletions were spotted on non-selective YES medium or on plates containing phloxine B, which indicates the proportion of dead cells in a colony. *cnp1-1* and *dis3-54* are temperature-sensitive strain controls.

Ard_Supplementary-Figure-3

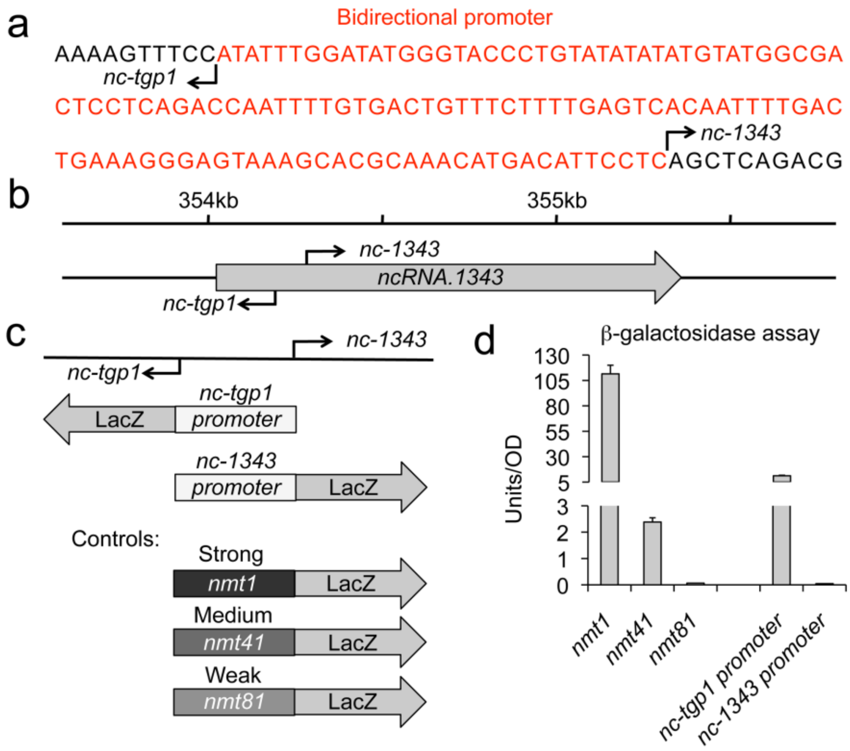

Supplementary Figure 3. A bidirectional lncRNA promoter upstream of *tgp1*⁺ preferentially drives transcription of *nc-tgp1*. (a) Transcription start sites (TSSs) for *nc-tgp1* and *nc-1343* were mapped by 5'RACE and indicated by arrows. Bidirectional non-coding promoter is underlined and displayed in red. (b) Schematic of *nc-tgp1* and *nc-1343* TSSs depicted within the annotated SPNCRNA.1343 locus. (c) Diagram of LacZ reporter under the control of the bidirectional promoter encoded within the *ncRNA.1343* locus (in both orientations) or control *nmt* promoters of varying strength (as indicated). (d) β-galactosidase assays from wild-type cells transformed with LacZ vectors.

Ard_Supplementary-Figure-4

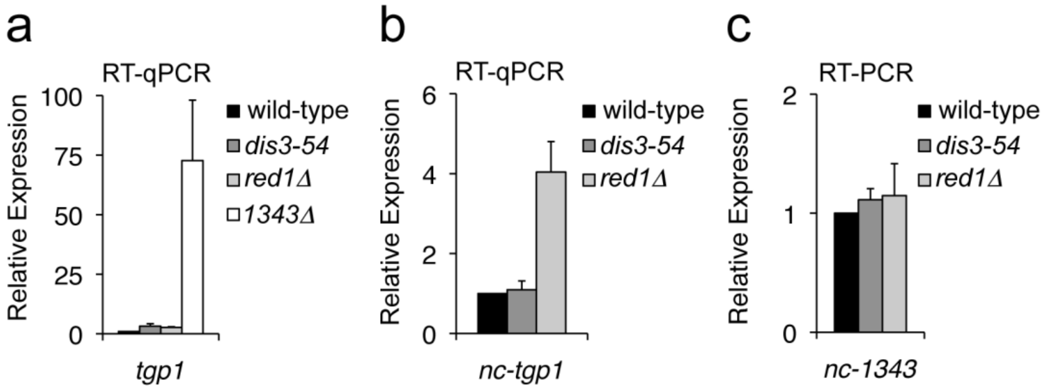

Supplementary Figure 4. Transcript levels at the *tgp1*$^{+}$ locus in Dis3 and Red1 mutants. RT-qPCR experiments to measure *tgp1*$^{+}$ (a), *nc- tgp1* (b), and *nc-1343* (c) transcript levels in wild-type, *dis3-54* and *red1Δ*. RNA levels quantified as relative to wild-type after normalization to *act1*$^{+}$; error bars indicate standard error from three independent experiments.

Ard_Supplementary-Figure-5

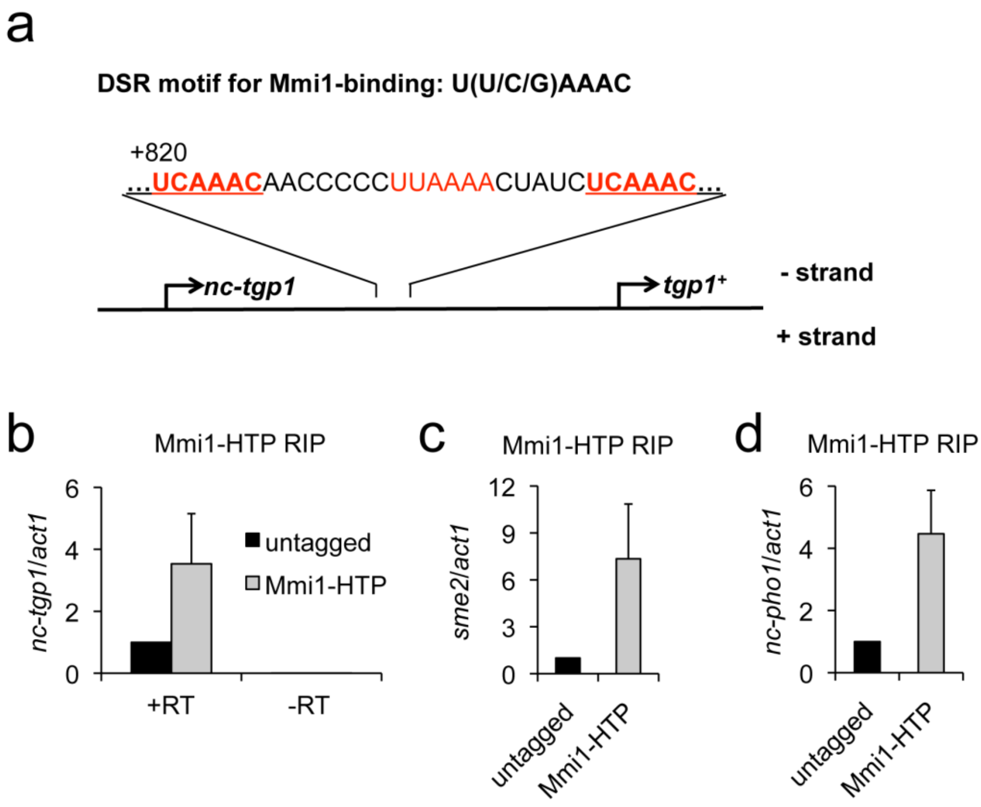

Supplementary Figure 5. *nc-tgp1* contains putative DSR sites for Mmi1-binding. (a) Schematic showing three putative DSR elements (two canonical: bold red text/underlined; one suboptimal: red text) embedded within the *nc-tgp1* transcription unit. Mmi1-HTP RIP and quantification by RT-qPCR for (a) *nc-tgp1*, (b) *sme2*$^{+}$, and (c) *nc-pho1* binding. Error bars indicate standard error from two independent experiments.

Ard_Supplementary-Figure-6

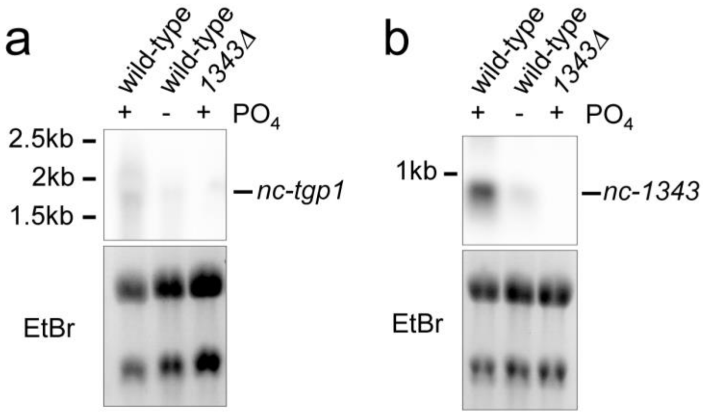

Supplementary Figure 6. Decreased *nc-tgp1* and *nc-1343* lncRNA levels following phosphate starvation. Northern analysis of *nc-tgp1* (a) and *nc-1343* (b) in the presence or absence of phosphate, and *1343Δ*□□□□□□ grown in the presence of phosphate.

Ard_ Supplementary-Figure-7

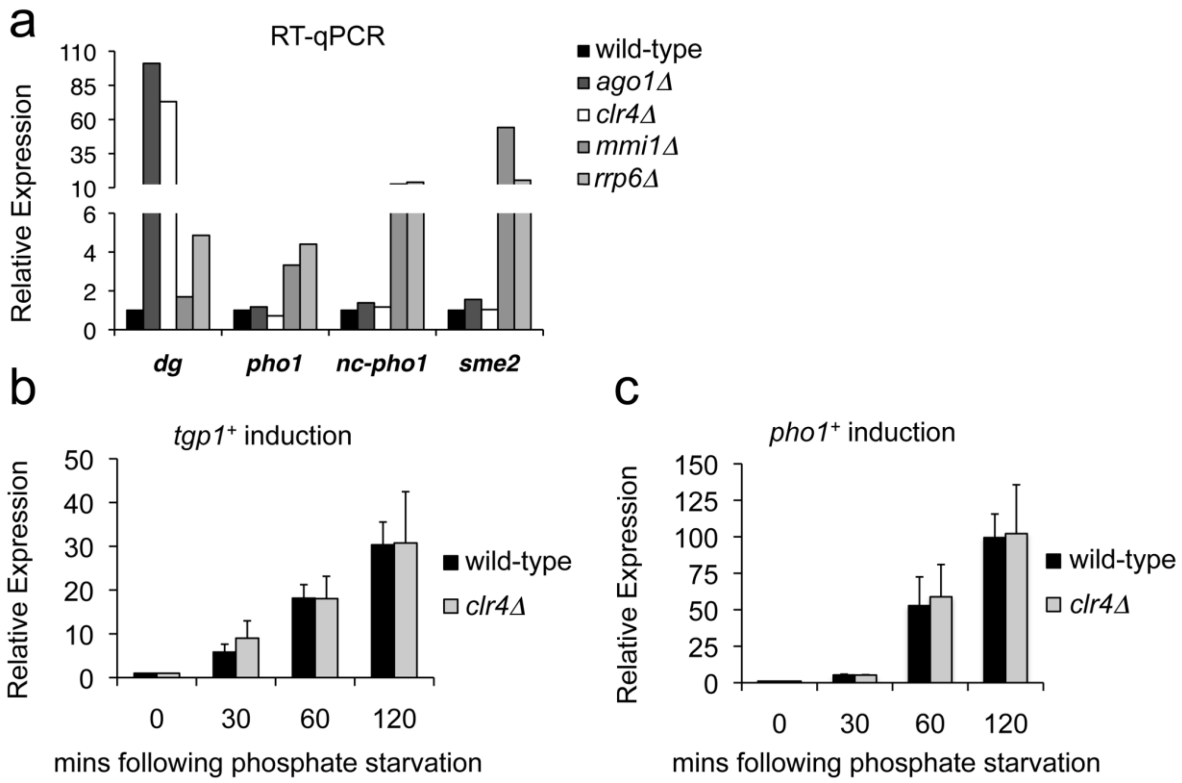

Supplementary Figure 7. Transcript levels in various mutants and conditions. (a) RT-qPCR experiments to measure *dg* (centromere repeats), *pho1*$^{+}$, *nc-pho1*, and *sme2*$^{+}$ transcript levels in wild-type, *ago1Δ*, *clr4Δ*, *mmi1Δ*, and *rrp6Δ*. RNA levels quantified as relative to wild-type after normalization to *act1*$^{+}$. RT-qPCR to measure (b) *tgp1*$^{+}$ and (c) *pho1*$^{+}$ mRNA induction kinetics following phosphate starvation in wild-type and *clr4Δ* cells. Error bars indicate standard error from two independent experiments.

Ard_Supplementary-Figure-8

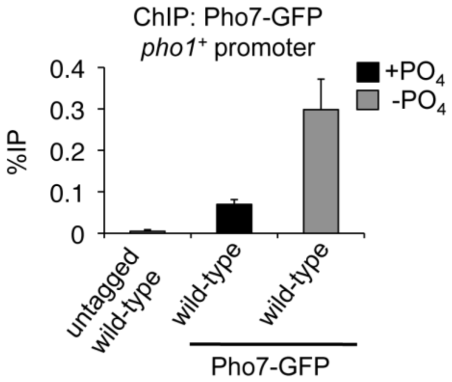

Supplementary Figure 8. Pho7-GFP accumulates at the *pho1*$^+$ promoter following phosphate starvation. The endogenous Pho7 transcription factor was C-terminally tagged with GFP in wild-type cells. GFP ChIP experiments were performed in the presence or absence of phosphate. An untagged strain was used as a negative control. Pho7-GFP levels at the *pho1*$^+$ promoter were measured by qPCR and quantified as %IP (ChIP/Input); error bars indicate standard error from three independent experiments.

Ard_Supplementary-Figure-9

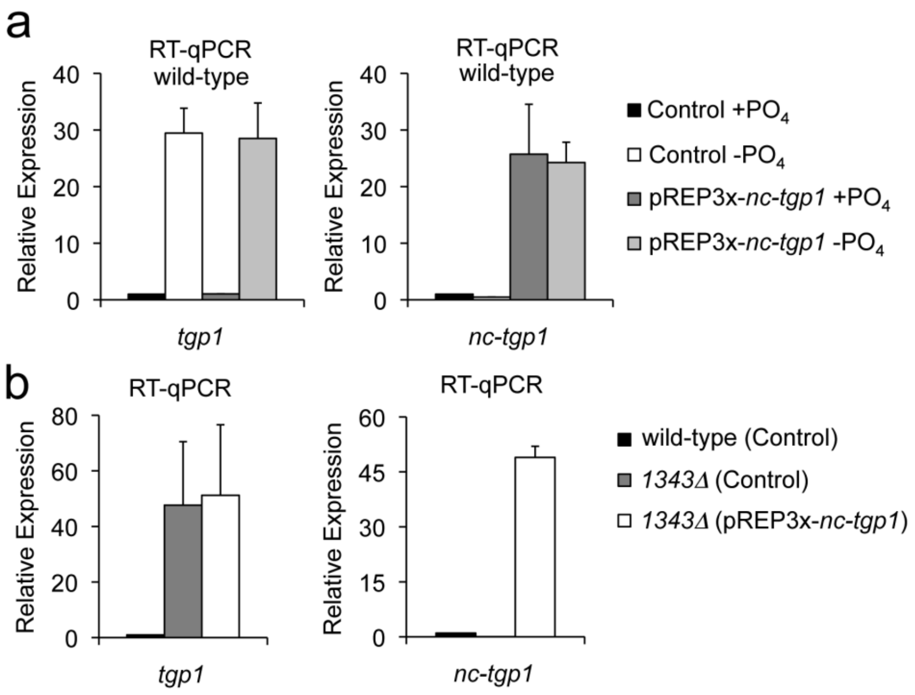

Supplementary Figure 9. *nc-tgp1* does not repress *tgp1*$^{+}$ *in trans*. RT-qPCR experiments to measure *tgp1*$^{+}$ mRNA and *nc-tgp1* lncRNA levels in (a) wild-type responding to phosphate availability, and (b) *1343Δ* cells, each transformed with an empty pREP3x vector (Control) or pREP3x vector containing *nc-tgp1* under the control of the strong *nmt1* promoter (pREP3x-*nc-tgp1*). Error bars indicate standard error from two independent experiments.

Supplementary Table 1. List of *S. pombe* strains used in this study.

Name	ID #	Genotype	Source
wild-type	143	“972 h-”	Lab stock
wild-type	1645	h+ ade6-210 arg3-D4 his3-D1 leu1-32 ura4-D18	Lab stock
wild-type	1646	h- ade6-210 arg3-D4 his3-D1 leu1-32 ura4-D18	Lab stock
rrp6Δ	7865	h+ rrp6Δ::kan ade6-210 ura4-D18 leu1-32	Lab stock
ago1Δ	8061	h+ ago1Δ::ura4 otr1R(SphI):ade6+ ura4-D18 leu1-32 ade6-M210	Lab stock
dcr1Δ	8146	h? dcr1Δ::KAN (G418R) ade6-210	Lab stock
swi6Δ	951	h90 swi6Δ::ura4 ura4-D18	Lab stock
clr4Δ	8435	h- clr4Δ::ura4 his7-366 ade6-210/216 leu1-32 ura4-D18	Lab stock
dis3-54	A1264	h+ dis3-54 ade6-216 leu1-32 arg3-D4	Lab stock
1343Δ::ura4$^+$	A9016	h+ SPNCRNA.1343Δ::ura4$^+$ade6-210 arg3-D4 his3-D1 leu1-32 ura4-D18	This study
1343Δ	A9032	h+ SPNCRNA.1343Δ ade6-210 arg3-D4 his3-D1 leu1-32 ura4-18	This study
tgp1Δ1343Δ	A9352	h+ 1343Δ tgp1Δ::ura4$^+$ ade6-210 arg3-D4 his3-D1 leu1-32 ura4-D18	This study
red1Δ	A9392	h90 red1Δ::kan leu1-32 ura4-D18 ade6-M210	Sugiyama, T.
mmi1Δ	A9393	h- mmi1Δ::kan leu1-32 mei4-P572	Sugiyama, T.
AΔ	A9520	h+ 1343AΔ ade6-210 arg3-D4 his3-D1 leu1-32 ura4-D18	This study
BΔ	A9522	h+ 1343BΔ□ade6-210 arg3-D4 his3-D1 leu1-32 ura4-D18	This study
nc-tgp1:ura4$^+$	A9523	h+ nc-tgp1:ura4+ ade6-210 arg3-D4 his3-D1 leu1-32 ura4-D18	This study
Pho7-GFP	A9827	h- pho7-GFP:NAT ade6-210 arg3-D4 his3-D1 leu1-32 ura4-D18	This study
Pho7-GFP/ 1343Δ::ura4$^+$	A9974	h- pho7-GFP:NAT 1343Δ::ura4$^+$ade6-210 arg3-D4 his3-D1 leu1-32 ura4-D18	This study
103Δ	A9027	h+ SPNCRNA.103Δ ade6-210 arg3-D4 his3-D1 leu1-32 ura4-18	This study
214Δ	A9028	h+ SPNCRNA.214Δ ade6-210 arg3-D4 his3-D1 leu1-32 ura4-18	This study
388Δ	A9029	h+ SPNCRNA.388Δ ade6-210 arg3-D4 his3-D1 leu1-32 ura4-18	This study
808Δ	A9030	h+ SPNCRNA.808Δ ade6-210 arg3-D4 his3-D1 leu1-32 ura4-18	This study
879Δ	A9031	h+ SPNCRNA.879Δ ade6-210 arg3-D4 his3-D1 leu1-32 ura4-18	This study
1443Δ	A9033	h+ SPNCRNA.1443Δ ade6-210 arg3-D4 his3-D1 leu1-32 ura4-18	This study
1556Δ	A9034	h+ SPNCRNA.1556Δ ade6-210 arg3-D4 his3-D1 leu1-32 ura4-18	This study
nmt1-nc-tgp1	B0200	h- nc-tgp1-promoter:nmt1-NAT ade6-210 arg3-D4 his3-D1 leu1-32 ura4-D18	This study
cnp1-1	6960	h- leu1-32 ura4-D18 cnp1Δ::ura4 lys1::cnp1-1	Lab stock
Mmi1-HTP	B0398	h+ mmi1-his6-TEV-ProA::KAN MX imr1R(NcoI)::ura4$^+$ ura4D-18 ade6-M216 leu1-32	Vasilieva, L.

Supplementary Table 2. List of oligonucleotides used in this study.

Name	Sequence
qAct1_F	GGTTTCGCTGGAGATGATG
qAct1_R	ATACCACGCTTGCTTTGAG
qSme2_F	AAACAAGGGAGGTAAACAGACTTAG
qSme2_R	GCATGCATATTCCGTCTTACAATAG
q1271.10c_F	CGCTTCGTATCTTTCTCTTTCC
q1271.10c_R	CCAGTCCTCTTCTTCGGTTGTA
q1271.09a_F (PP: 1*)	TCGGTTGGAATGTTCTAATCAATAC
q1271.09a_R (PP: 1*)	AGACCGGTGATCAAACAATATTTAG
q1271.09b_F (PP: 2*)	TGAAGTAGTTAGACAGGTTAGCGA
q1271.09b_R (PP: 2*)	CTTGTCGTCCAACTTCTCTTCATC
qnctgp1c_F (PP: 3; pro*)	GGCAGTAAATCTATCTGTAGCGAGT
qnctgp1c_R (PP: 3; pro*)	TACACGGTAAATGTCAAGTCTGCTA
qnctgp1b_F (PP: 4*)	CTGACAAACCAATTATCCCTACACG
qnctgp1b_R (PP: 4*)	GTATTACGATTTGGCAACCTCATCC
qnctgp1a_F (PP: 5*)	TTAAATGCTGCACTCACATACTGAC
qnctgp1a_R (PP: 5*)	ACTCTCCCTTGGGTTCATTTGATTA
qncRNA1343_F (PP: 6*)	ATACAGACGTGTGGATTGCAA
qncRNA1343_R (PP: 6*)	CCTCTTCTATACGCAATCAATGTC
q1271.08c_F	TTCAAGGAGCATTTCAATTCTAAAC
q1271.08c_R	TATGTATCGTTAGTTATGCCTCGTG
qMug96_F	CATCCTATGTTTATTTGTCTGTTGC
qMug96_R	CTCATGATGGTCCTTAAACCTATTG
qPho1_F	CTTTGGACCCTCTAATACATCCGAT
qPho1_R	AAGAGTGTCAAAGTTCTGGATACCA
qncPho1_F	ATGATGTTTGAGATTTACGGGAAGT
qncPho1_R	TTCTGTAAATGTGTCCCGAACCAAA
qDg_F	AATTGTGGTGGTGTGGTAATAC
qDg_R	GGGTTCATCGTTTCCATTCAG
lacZ_1_F	TACTACGTCGACCGACTGACCTCAAACCAAACAGCA
lacZ_1_R	TACTACCTGCAGTCACTAATGTCATACTCGGCTTGAG
lacZ_2_F	TACTACCTGCAGCGACTGACCTCAAACCAAACAGCA
lacZ_2_R	TACTACGTCGACTCACTAATGTCATACTCGGCTTGAG
nc-tgp1_SalI_F	TACTACGTCGACCATATCCAAATATGGAAACT
nc-tgp1_XmaI_R	TACTACCCCGGGCTGCCGACTTACAAGTCTCG

*****(PP: primer pairs; pro: promoter)
